# Military-civilian cooperative emergency response to infectious disease prevention and control in China

**DOI:** 10.1186/s40779-016-0109-y

**Published:** 2016-12-30

**Authors:** Hui Ma, Ji-Ping Dong, Na Zhou, Wei Pu

**Affiliations:** Health Bureau, Logistics Support Department of Central Military Commission, Chinese PLA, Beijing, 100842 China

**Keywords:** Infectious disease, Emergency response, Military-civilian cooperation

## Abstract

In recent years, the incidence of severe infectious diseases has increased, and the number of emerging infectious diseases continues to increase. The Chinese government and military forces have paid a great deal of attention to infectious disease prevention and control, and using military-civilian cooperation, they have successfully prevented numerous severe epidemic situations, such as severe acute respiratory syndrome (SARS), influenza A (H1N1), avian influenza H5N1 and H7N9, and Ebola hemorrhagic fever, while actively maintained public health, economic development, and national construction. This paper focuses on the mechanisms of the military-cooperative emergency response to infectious diseases--the joint working mechanism, the information-sharing mechanism, the research collaboration mechanism, and the joint disposal mechanism--and presents a sorted summary of the practices and experiences of cooperative emergency responses to infectious diseases. In the future, the Chinese military and the civilian sector will further strengthen the cooperative joint command system and emergency rescue force and will reinforce their collaborative information-sharing platform and technical equipment system to further improve military-civilian collaborative emergency infectious diseases disposal, advance the level of infectious disease prevention and control, and maintain public health.

## Background

In recent years, the prevalence of severe infectious disease has increased, and the number of emerging infectious diseases continues to increase [1]. The Chinese government and military forces have been highly focused on infectious disease prevention and control, and through military-civilian cooperation, they have successfully prevented numerous severe epidemic situations, such as sudden acute respiratory syndrome (SARS), influenza A (H1N1), avian influenza H5N1 and H7N9, and Ebola hemorrhagic fever, while actively maintaining public health, economic development, and national construction [2–9]. This article introduces military-civilian joint prevention and control as well as prevention and control experiences to provide a basis for reference for future infectious disease prevention and control.

The military-civilian collaborative emergency response to infectious diseases refers to the integration of military and government strengths and resources during a certain time period and range, according to the principles of unified command, resource sharing, and close cooperation, in order to solve and jointly respond to epidemic outbreaks of infectious diseases, minimize harm to human health, and maintain social stability [10].

In 2004, the Chinese government disseminated “the Law of the PRC on the Prevention and Treatment of Infectious Diseases,” which clearly defined many aspects of infectious diseases, such as epidemic reports, epidemic situation control, medical rescue, supervision and management, guarantee measures, and legal liability, among others; this law provides a legal basis for effectively preventing and eliminating infectious disease occurrence and prevalence and guarantees the health and hygienic safety of the public. The general principle of this law clearly stipulates that military prevention and control of infectious diseases should be consistent with the law and relevant national provisions [11]. Moreover, “Regulations on Preparedness for and Response to Emergent Public Health Hazards” was also formulated, which clearly stipulates that the involvement of the People’s Liberation Army’s (PLA’s) medical and health institutions should be consistent with these regulations and relevant military provisions during emergency treatment and health incidents [12]. The military formulated “the Regulations of the PLA’s prevention and control of infectious diseases” in 2008 and the “Emergency Plan for Military Disposal of Emergency Public Health Events” in 2009, as well as the “Military-civilian Cooperation Mechanism of Prevention and Control for Infectious Diseases” in 2006 and the “Military-civilian Cooperation Mechanism of Emergency Disposal for Emergency Public Health Events” in 2008. All of these laws and regulations have laid a solid institutional foundation for the military-civilian collaborative emergency response to infectious diseases [13].

## The operational mechanism of the military-civilian collaborative emergency response

Under the unified leadership and overall deployment of the nation and the military, the collaborative emergency response mechanism of military-civilian integration and efficient linkage has been established, and it develops a pattern of complementary advantages and efficient collaboration as well as rapidly improves national and military infectious disease prevention and control [14–16]. For example, five cases of Zika virus infection imported from Venezuela emerged in Guangdong Province in February of 2016. The Academy of Military Medical Sciences immediately initiated the emergency joint mechanism and acquired samples from Guangzhou No.8 People’s Hospital. The two Zika virus strains were isolated from blood and urine samples acquired from the two patients. The urine test was the first successful isolation of Zika from a sample. The gene sequence of the first imported Zika virus was also determined through the military-civilian collaboration.

### Joint working mechanism among departments

The nation has established a joint working mechanism among different departments, and this mechanism indicates that each department is responsibility for itself, coordinates with the other departments, and jointly implements the prevention measures under central unified leadership. The military joint prevention and control came into effect by the unified leading, joint supervision, persevering management, and workforce formation of medical treatment, health, publicity, and rear service among multiple departments. The military and the civilian sector cooperated often and closely, establishing the joint working mechanism; for example, military experts were appointed to participate in leading expert groups for joint prevention and controlled response to emergency events and in the formulation of relevant policies, regulations, and professional documents. Meanwhile, the military’s working progress was reflected over time, and the military-civilian coordination and communication mechanism was constructed.

### Nationwide epidemic data-sharing mechanism

An epidemic information report and sharing mechanism has been established. Multiple tasks, such as information report development, joint notification and report release, and joint research on case diagnoses and epidemic announcements, are undertaken daily. The epidemic data from local disease control and prevention institutions are shared nationwide in a timely manner using similar procedures and the epidemic report channel, which establishes the collaborative surveillance and forewarning mechanism that discovers early epidemic outbreaks and provides strong technical support so that prompt and effective military-civilian control measures can be taken.

### Research collaboration mechanism of the military-civilian institutions

Once an epidemic situation has occurred, the military immediately initiates the emergency research mechanism, sets up rapid research channels, and deploys emergency research tasks to scientific research institutions. In the meantime, the national and military disease control and prevention systems immediately integrate scientific research, organize scientific problem-solving measures, collaboratively establish research and development engineering projects, and jointly develop detection reagents and drug-resistant vaccines in response to outbreaks and the prevalence of infectious diseases. These actions can prevent and control epidemic situations.

### Joint disposal mechanism at military-civilian levels

According to the national statement on epidemic information and the national strategy for prevention and control, epidemic prevention and control are jointly deployed at all military-civilian levels, the infection source and close contacts are traced over time, medical quarantine is carefully carried out, and personnel screening and medical observation are put into practice. Military experts and military disease control and prevention institutions at all levels, who are obligated to execute these plans due to national requirements, should actively participate, following the laboratory test index, outbreak investigation program, and clinical treatment standards. In addition, the military is also required to participate in the emergent disposal of local epidemic outbreaks; successful past experiences that resulted in preferable public effects can be attributed to the army.

## Successful experiences and practices of the military-civilian cooperative emergency response

The government and the military have always attached great importance to infectious disease prevention and control. After the SARS epidemic, the country rapidly intensified construction of its public health system, effectively controlled the spread of several severe epidemic situations, and showed the strong capability of the military-civilian collaborative emergency response, relying heavily on the national military system. This was especially evident when the Ebola epidemic outbreaks occurred in West Africa in 2014; China was the first to lend a helping hand, sending approximately 1,200 military-civilian medical and health personnel to carry out laboratory tests, make diagnoses, provide treatment and public health training, and build a biological safety laboratory for the affected countries. This is the largest healthcare foreign-aid action of a military-civilian collaborative emergency response since the founding of new China [17]. The main successful experiences and practices are as follows [18–21].

### Normalization of the military-civilian collaborative emergency command mechanism

The primary principles of the military-civilian collaborative emergency response are unified command, graded responsibilities, and resident management. By establishing a unified national and military command organization, the comprehensive coordinative command of the military, police, and people was implemented; scientific and effective emergency decisions were made; institutional and functional orientations at all levels were defined; emergency power from all fields was motivated and coordinated for joint participation; unblocked government decree was ensured; and an advantageous configuration of resource integration was achieved.

### Institutionalization of military-civilian collaborative emergency organization and operation

To effectively address severe epidemics, the government and military formulated several laws, regulations, policies, strategic development plans, joint action programs, emergency safety measures, and various types of operable emergency predetermined plans; clearly defined the division of duties and transactions; established an assessment mechanism for military-civilian joint epidemic situations and a docking mechanism for military-civilian relevant departments; and jointly improved the ability to respond to infectious disease emergencies.

### Informatization of military-civilian collaborative management means

By using network and communication technologies, an information-sharing mechanism was established and characterized by directional coverage and frequent communication, and a military-civilian collaborative management platform for emergency rescue that can acquire real-time epidemic information, quick responses, and integrated linkage of emergency epidemic situations was built. After the Wenchuan earthquake in 2008, an epidemic reporting system was established based on the mobile network, which greatly improved the capability of the military-civilian collaborative emergency response.

### Specialization of military-civilian collaborative prevention and control

Military emergency prevention and control provides important support for not only the military itself but also national missions, such as emergency disposal during public health emergency events, major activity security, and major domestic and overseas disaster rescue. Since 2009, the Military Emergency Medical Rescue Team, the Emergency Epidemic Prevention Team, the anti-Nuclear, Chemical, and Biological (anti-NBC) Medical Field Rescue Team; and the Veterinary Health, Prevention and Control Team have all been included in the national emergency system, which can greatly enhance the capacities of military-civilian collaborative emergency rescue, prevention, and control.

## Past achievements and future prospects

In recent years, the national health department has taken the lead and constructed multi-department cooperation, including a health emergency work mode for joint prevention and control that involves the military and coordinates the linkage and efficient decision-making across departments and regions [22]. A series of laws, regulations, and pre-arranged plans were formulated; an effective coordinative command mechanism for military-civilian epidemic situations was perfected; an accurate and quick information reporting system was optimized; and military-civilian collaborative professional emergency disposal teams were cultivated, all of which led to outstanding achievement in preventing and controlling SARS, H1N1, and several other severe infectious diseases [23] (See also Table 1). The responses to H1N1 in 2009 and to avian influenza H7N9 in 2014 highlighted the importance of military-civilian joint prevention and control. Test reagents were co-developed in record time; efficient medicine and a specific vaccine for H1N1 were developed and prepared, effectively preventing the prevalence of H1N1 in China. A Peramivir and sodium chloride injection was successfully developed and prepared, which played an important role in treating severe H7N9 cases. During the prevention and control of Ebola hemorrhagic fever in Western Africa, military joint national medical rescue contributed significantly to fighting the outbreak in Sierra Leone and Liberia [24, 25].

**Table 1:** Infectious disease emergency events and the military-civilian cooperative response

Infectious disease events	Year	Military-civilian contributions
SARS	2003	First isolation of the virus; sequencing of the entire virus genome; development of rapid diagnosis assays; establishment of SARS-designated hospitals and treatment of the patients
H5N1 avian flu	2004 to 2005	Large-scale industrial development of the Tamiflu equivalent, Oseltamivir (brand name KeWei)
H1N1 flu	2009	Confirmation of first input H1N1 infected case; development of rapid diagnosis assays and RT-PCR; development of Peramivir Trihydrate and H1N1 flu vaccine
H7N9 flu	2013 to 2016	Development of rapid diagnosis assays and RT-PCR; development of Peramivir and sodium chloride injection
Ebola hemorrhagic fever	2014 to 2015	Deployment of testing teams and mobile hospitals; treatment of patients and training of the health care workers; establishment and operation of biosafety III laboratory; development of Ebola drug MIL 77 and vaccine
Zika virus infection	2015 to 2016	Confirmation of first input Zika virus infection case; isolation of Zika virus strains; gene sequencing of the first imported Zika virus; development of rapid diagnosis assays

Although the Chinese military-civilian emergency collaboration has accomplished certain achievements regarding prevention and control of infectious diseases, there are still many problems with the collaborative organization and command, professional power construction, equipment performance level, and emergency theory research, which can be solved by establishing a deep civil-military integration mechanism [26]. Chinese president Jin-ping Xi, who is also the general secretary of the Communist Party of China (CPC) Central Committee and the chairman of the Central Military Commission, has called for further integration of military and civilian undertakings that could cover multiple areas and generate high returns, and he has urged military and local authorities to advance their work while considering the overall development of the country. Civilian-military integration has made the synchronized development of economic and national defense capabilities possible. The PLA should play an active role in local economic and social development and contribute to wellbeing of the public through actual deeds, including the military-civilian cooperative emergency response to infectious disease prevention and control.

## Conclusions

This paper focuses on the mechanisms of the military-cooperative emergency response to infectious diseases--the joint working mechanism, the information-sharing mechanism, the research collaboration mechanism, and the joint disposal mechanism--and presents a sorted summary of the practice and experience of the cooperative emergency response to infectious diseases. The Chinese military and the civilian sector will further strengthen the military-civilian collaborative joint command system and emergency rescue, reinforce the military-civilian collaborative information-sharing platform and technical equipment system, develop theoretical research on the military-civilian collaborative emergency response, enhance the training and modular exercises of the emergency teams, and strengthen the measures for epidemic information sharing, technical cooperation, disposal linkage, and integral prevention and control, which may further improve military-civilian collaborative emergency disposal, promote infectious disease prevention and control, and maintain public health.

## Abbreviations

CPC: Communist party of China
NBC: Nuclear, chemical and biological
PLA: People’s liberation army
SARS: Severe acute respiratory syndrome
